# A Reporter Gene Assay for Measuring the Biological Activity of PEGylated Recombinant Human Growth Hormone

**DOI:** 10.3390/molecules30030669

**Published:** 2025-02-03

**Authors:** Shaowang Hu, Xiaoming Zhang, Yi Li, Jing Li, Yingwu Wang, Chenggang Liang

**Affiliations:** 1 School of Life Science, Jilin University, Changchun 130012, China; hu17606194353@163.com; 2 National Institutes for Food and Drug Control, Beijing 100061, China; zhangxm@nifdc.org.cn (X.Z.); liyi2016@nifdc.org.cn (Y.L.); li_jing@nifdc.org.cn (J.L.)

**Keywords:** PEGylated recombinant human growth hormone (PEG-rhGH), quality control, reporter gene assay (RGA), HepG2/IGF-1 cell line, positional isomers, bioactivity assay

## Abstract

PEGylated recombinant human growth hormone (PEG-rhGH) has garnered significant interest in growth hormone research due to its prolonged half-life and improved patient compliance. An accurate evaluation of its biological activity is critical for ensuring the quality of PEG-rhGH-based therapeutics. In this study, we established an in vitro bioactivity assay using a reporter gene method based on the HepG2/IGF-1 cell line. Key assay parameters, including the initial concentration of PEG-rhGH, serial dilution ratios, cell density, and incubation time, were systematically optimized to generate robust dose–response curves. The assay demonstrated high sensitivity, precision, and reproducibility across multiple batches of PEG-rhGH. The validation results showed an excellent correlation with traditional in vivo animal studies and the Nb2-11 cell proliferation assay, highlighting its suitability for quality control. Furthermore, we developed an ion exchange chromatography (IEC) method to separate five positional isomers of PEG-rhGH, revealing significant differences in bioactivity depending on the PEG modification site. This study demonstrates that the optimized reporter gene assay is not only effective for quality control of PEG-rhGH but also serves as a valuable tool for evaluating and optimizing PEGylated long-acting growth hormone therapeutics.

## 1. Introduction

Human growth hormone (hGH) is a protein hormone that is secreted by acidophilic cells of the human pituitary gland [1]. It consists of 191 amino acids, with a molecular weight of 22,125 Da, containing two disulfide bonds but lacking glycosylation sites [2]. Since 1982, recombinant human growth hormone (rhGH) has been approved by the FDA for the treatment of growth hormone deficiency (GHD) and has been widely used to promote growth and development in children [3,4,5,6]. However, due to the short half-life of rhGH in the bloodstream, the therapeutic effect largely depends on accurate diagnosis, appropriate dosing regimens, and patient adherence [7]. In adolescents, the issue of adherence is particularly severe, with non-compliance rates as high as 77%, significantly affecting treatment outcomes [8,9]. Despite numerous improvements in injection devices, formulations, and storage methods, many patients still face adherence challenges due to the need for daily injections [10].

To extend the half-life of the drug and reduce the injection frequency, PEGylated recombinant human growth hormone (PEG-rhGH) was developed by covalently attaching large PEG molecules to the free amino groups of rhGH. This modification increases the molecular weight and volume of the compound, reduces the renal clearance, significantly prolongs the drug’s half-life in vivo [11], and reduces the injection frequency from once daily to once weekly, thereby markedly improving patient adherence. Jintrolong^®^ is the first long-acting PEGylated rhGH formulation that is globally approved for the treatment of GHD, utilizing branched PEG molecules that are covalently bound to the amino groups of rhGH to extend the half-life in the body. Studies have shown that this formulation does not lead to common early adverse effects such as lipoatrophy at the injection site [12,13]. rhGH has 10 sites that can be modified by PEGylation, which are the free N-terminal amino group and the free ε-amino groups of lysine residues. This leads to variability in modification sites. This heterogeneity in PEGylation can impact the biological activity and potency of the drug. Traditional animal model methods are unable to effectively distinguish the activity differences among PEG-rhGH variants with different modification sites, thus failing to comprehensively reflect the impact of modification sites on the therapeutic efficacy [14].

In addition to Jintrolong^®^, several other long-acting growth hormone products have been marketed or are under clinical investigation. For example, Lonapegsomatropin [15], the first prodrug utilizing TransCon technology [16,17], was approved in the United States in 2021 for the treatment of pediatric GHD [18,19,20]. Somapacitan, a long-acting growth hormone that is extended through fatty acid modification, was approved in 2020 for adult GHD treatment and subsequently in Japan and Europe in 2021 [21]. Eftansomatropin alfa, developed by South Korea’s Genexine [22] using its innovative Fc fusion protein technology platform (hyFc^®^), has completed phase III clinical trials and is preparing for market launch [23,24]. The introduction of these products provides more diverse treatment options for patients but also presents challenges regarding the variability in bioactivity among positional isomers with different modification sites.

The in vivo animal method is considered the gold standard for measuring the biological activity of recombinant human growth hormone (rhGH). However, this method requires hypophysectomy via the parapharyngeal route. During the procedure, the pituitary gland must be completely removed while avoiding damage to surrounding nerves and brain tissues, making the surgery extremely challenging. Skilled technicians can achieve approximately 40% surgical success rates, and the method requires a significant number of experimental animals [25]. Moreover, the entire testing process takes about two months.

In the 43rd edition of the *United States Pharmacopeia–National Formulary* (USP-NF), the in vivo bioassay for recombinant human growth hormone (rhGH) has been removed, retaining only the in vitro cell culture method based on the rat lymphoma cell line Nb2-11. The *European Pharmacopoeia* (EP) recommends using validated bioassays to determine the activity of recombinant human growth hormone (rhGH), including validated in vitro cell methods and in vivo methods, to ensure the reliability and accuracy of rhGH potency determination [26]. Similarly, the 2025 draft version of the *Chinese Pharmacopoeia* has replaced the in vivo animal method with the Nb2-11 cell proliferation assay for measuring rhGH biological activity.

The Nb2-11 cell assay relies on the expression of the prolactin receptor, which shares a common ancestral gene with the growth hormone receptor. Notably, human prolactin and interleukin-2 can also promote the proliferation of Nb2-11 cells, thereby compromising the specificity of the assay [27]. This method cannot accurately reflect the interaction between rhGH and the growth hormone receptor in vivo. Additionally, it is time-consuming, requiring at least 30 h from cell seeding to result acquisition.

To address these limitations, our research team established an in vitro bioactivity assay for rhGH based on the HepG2/IGF-1 cell line. This cell line stably expresses the growth hormone receptor (GHR), *STAT5B*, and the *3H-IGF-1-P2-Luc* reporter gene, enabling the assessment of rhGH bioactivity by measuring luciferase expression levels [28]. According to USP 1032, evaluating the dose–response relationship with the 4PL model provides more comprehensive information about data behavior and reduces the risks associated with applying a linear model to the central linear portion of the curve. Since the linear model contains relatively fewer data points in the middle section, any unpredictable shifts over time could significantly impact the experimental results. Therefore, the 4PL model was selected for this study. In this study, we utilized the HepG2/IGF-1 cell line to develop a reporter gene assay (RGA) for measuring the in vitro activity of PEG-rhGH, optimized through experimental conditions

We further evaluated the biological activity of multiple batches of PEG-rhGH using both animal models and cell-based assays. Ion chromatography was employed to separate five positional isomers from the PEG-rhGH samples, and bioactivity assessments revealed significant differences in activity depending on the modification site. This study provides an effective tool for the quality control of PEG-rhGH products and serves as a means to monitor activity differences among PEG-rhGH variants with different modification sites. These findings highlight the critical role of modification sites in drug efficacy and offer valuable insights for the development of other long-acting biologics.

## 2. Results

### 2.1. Optimization of Reporter Gene Assay

**Figure 1:**
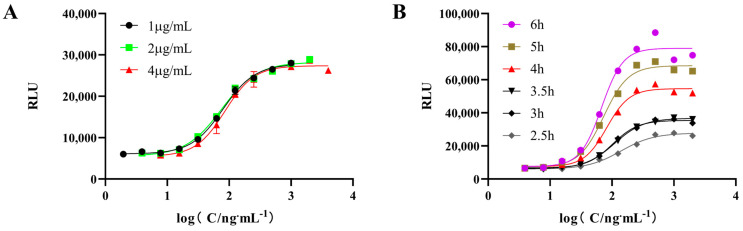
Optimization of the reporter gene assay. (**A**) Dose–response curves after adding different working concentrations of PEG-rhGH; (**B**) dose–response curves at different stimulation times.

To achieve a typical sigmoidal dose–response curve, we first optimized the concentration of PEG-rhGH. HepG2/IGF-1 cells were treated with gradient-diluted PEG-rhGH standards at initial concentrations of 1.0, 2.0, and 4.0 μg/mL. In Figure 1A, the X-axis represents the logarithm of PEG-rhGH concentration (log[PEG-rhGH], ng/mL), while the Y-axis represents the relative luminescence units (RLU). For 1 μg/mL, 2 μg/mL, and 4 μg/mL concentrations, the S/N ratios were 4.6, 5.1, and 5.0, respectively, while the R^2^ values were 0.997, 0.995, and 0.988, respectively. Although the signal-to-noise ratio for 4 μg/mL was comparable to that of 2 μg/mL, the higher R^2^ value that was observed at 1 μg/mL suggested better curve fitting. Considering the balance between signal intensity, noise levels, and curve fitting accuracy, 2 μg/mL was selected as the optimal concentration to ensure robust and reproducible results. The dose–response curve exhibited a typical sigmoidal shape at 2.0 μg/mL, with an R^2^ value of 0.995 for the fitted curve.

Next, we optimized the incubation time by testing six intervals: 2.5, 3, 3.5, 4, 5, and 6 h. As shown in Figure 1B, the dose–response curves displayed clear sigmoidal shapes with distinct upper and lower saturation phases at all time points. The signal-to-noise ratios for all intervals exceeded 3. The R^2^ values for the fitted curves at each time point were 0.9825, 0.9921, 0.9926, 0.9990, 0.9956, and 0.9905, respectively. Among these, the 4 h incubation time yielded the highest R^2^ value of 0.9990, indicating the best curve fitting and data reliability. In addition, at 5 and 6 h, a decline in signal was observed in the saturation phase, suggesting potential over-incubation effects. Therefore, 4 h was selected as the optimal stimulation time for this assay to ensure accuracy and reproducibility.

### 2.2. Validation of HepG2/IGF-1 Cell-Based Reporter Gene Assay (RGA)

#### 2.2.1. Accuracy, Precision, and Linearity of RGA

According to ICH Q2 (R2) and ChP 9401, accuracy refers to the closeness of a measured value to the true or reference value, while linearity describes the ability to produce results that are directly proportional to the theoretically expected values. In this study, PEG-rhGH samples were prepared at initial concentrations of 1 μg/mL, 1.42 μg/mL, 2 μg/mL, 2.82 μg/mL, and 4 μg/mL. A separate 2 μg/mL PEG-rhGH sample was prepared for in-house control. Each concentration was measured four times by two analysts.

**Figure 2:**
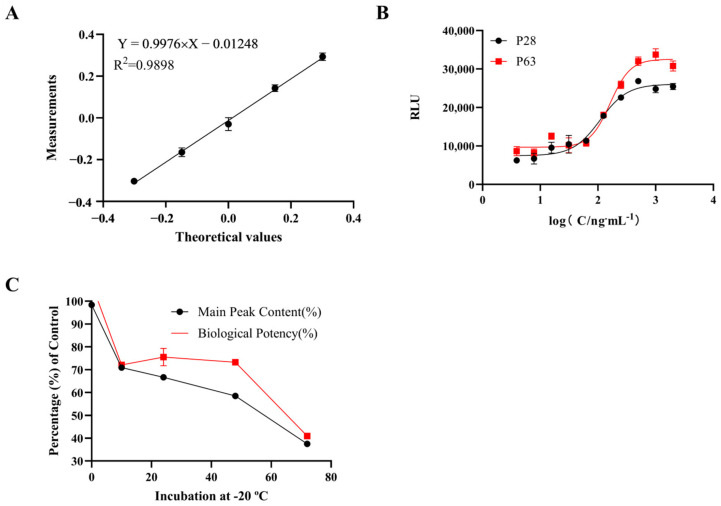
Validation of the HepG2/IGF-1 cell-based RGA. (**A**) Linearity of the RGA. (**B**) Stability of the RGA. (**C**) Stability and potency assessment of PEG-rhGH.

As shown in Table 1, the relative bias ranged from −6.6% to −0.4%, and the geometric coefficient of variation (GCV%) across the five potency levels ranged from 2.1% to 7.4%. Additionally, the correlation coefficient of the linear regression equation was 0.9898 (Figure 2A), indicating that the method demonstrated excellent accuracy, intermediate precision, and linearity.

#### 2.2.2. Stability of RGA

The stability of the HepG2/IGF-1 cell line was evaluated at passage 28 and passage 63. As shown in Figure 2B, both passages exhibited a signal-to-noise ratio of 4.9 and an R^2^ value exceeding 0.98 for the four-parameter curve fitting. These findings confirm that the HepG2/IGF-1 cell line maintains consistent stability across different passages.

#### 2.2.3. Stability and Potency Assessment of PEG-rhGH Using RGA

**Figure 3:**
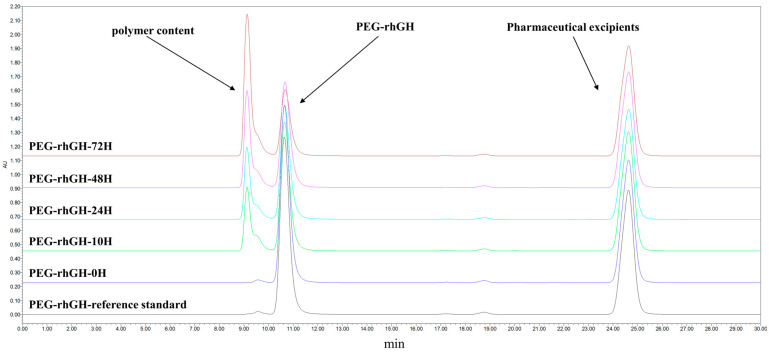
Chromatogram of polymer content determination for incubated samples at −20 °C.

The polymer content refers to the proportion of higher-order aggregates that are formed in PEG-rhGH formulations. PEG-rhGH formulations were incubated at −20 °C for 0, 5, 10, 24, 48, and 72 h to evaluate their stability under low-temperature conditions. The polymer content of the samples was analyzed using Size Exclusion Chromatography–High-Performance Liquid Chromatography (SEC-HPLC). As shown in Figure 3, the polymer content increased from 1.55% at 0 h to 62.28% at 72 h. The bioactivity of the incubated samples was subsequently assessed using RGA. As shown in Figure 2C, compared to the PEG-rhGH reference standard, both the bioactivity and main component content decreased with prolonged incubation times. These findings indicate that the RGA method can effectively assess the impact of low-temperature exposure on PEG-rhGH by detecting reductions in bioactivity associated with a decrease in the main component content.

### 2.3. In Vivo and In Vitro Potency of Multiple Batches of PEG-rhGH

To evaluate the feasibility of replacing the classic in vivo animal assay and the Nb2-11 cell proliferation assay with the RGA method, the potency of nine batches of commercial PEG-rhGH formulations injections was assessed using the in vivo bioassay, the newly developed RGA, and the Nb2-11 cell proliferation assay. The biological activity data are presented in Table 2.

**Figure 4:**
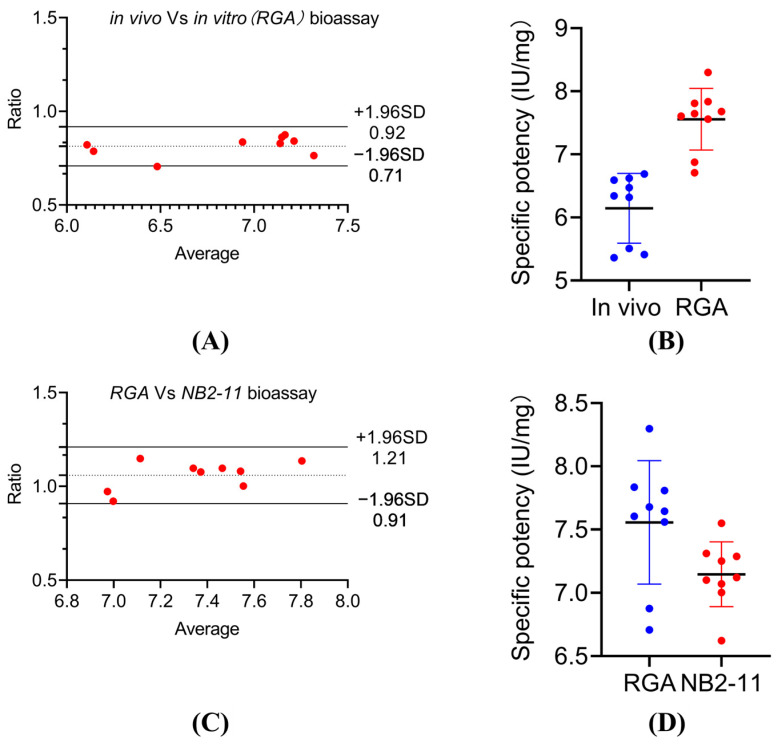
Comparison of in vivo and in vitro potency determination for PEG-rhGH using RGA and Nb2-11 cell proliferation assays: (**A**) Bland–Altman analysis comparing in vivo and in vitro (RGA) potency determination; (**B**) specific potency (IU/mg) of PEG-rhGH measured by in vivo and in vitro (RGA) bioassays; (**C**) Bland–Altman analysis comparing RGA and Nb2-11 potency determination; (**D**) specific potency (IU/mg) of PEG-rhGH measured by RGA and Nb2-11 bioassays.

As shown in Figure 4A,B, a Bland–Altman analysis demonstrated strong agreement between the RGA and in vivo assay results, with an average ratio of 81% and all data points being within the 95% agreement interval (71–92%). Similarly, Figure 4C,D indicate that the RGA showed excellent consistency with the Nb2-11 assay, with an average ratio of 106% and all data points falling within the 95% agreement interval (91–121%).

### 2.4. Separation, Enrichment, and Peak Identification of PEG-rhGH Isomers

**Figure 5:**
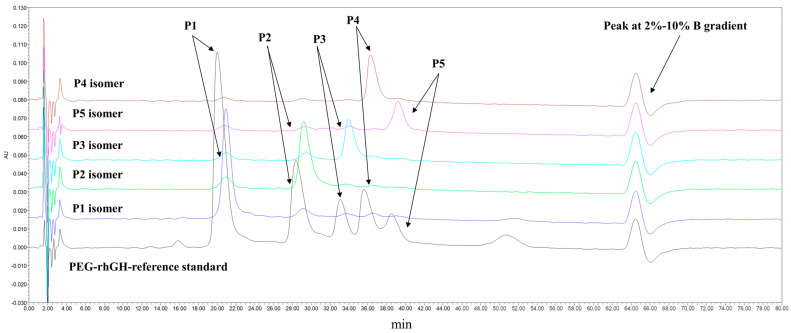
Chromatographic separation and collection of PEG-rhGH isomers.

Ion exchange chromatography (IEC) is a powerful technique that is used for separating components based on their charge differences. As shown in Figure 5, since PEG-rhGH isomers may have different charges, a method for separating different isomers of PEG-rhGH samples was successfully developed using IEC-HPLC. The chromatogram (black lines) reveals that the PEG-rhGH sample consists of at least five components with distinct retention times and proportions: 46%, 22%, 10%, 9%, and 13%, respectively. These components were labeled as P1, P2, P3, P4, and P5, and as shown in Figure 5, the last peak consistently appears in all chromatograms; this peak is a result of changes induced by the gradient elution. Following this method, the P1–P5 isomers were collected and analyzed. The retention times of the P1~P5 isomers were consistent with the P1~P5 contents of the PEG-rhGH reference standard, ensuring the accurate collection of the components.

### 2.5. Quantification of Collected P1~P5 Isomers

**Figure 6:**
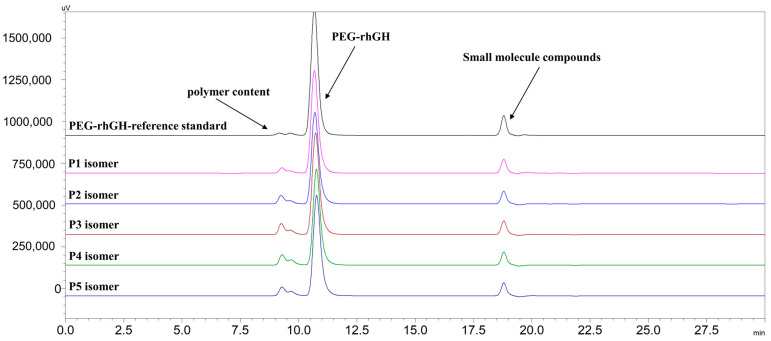
SEC chromatographic separation of isomers and PEG-rhGH reference standard.

To quantify the different isomers for subsequent potency assays, PEG-rhGH was used as a reference to quantify isomers P1–P5. The quantification was performed using the area normalization method, where the content of isomers P1~P5 was calculated based on the main peak area of the reference standard (0.985 mg/mL). In Figure 6, the separated PEG-rhGH isomers are represented by distinct colored curves. The quantification results, summarized in Table 3, indicated that the concentrations of isomers P1~P5 were 0.958, 0.864, 0.968, 0.936, and 0.940 mg/mL, respectively.

### 2.6. Molecular Weight Determination

**Figure 7:**
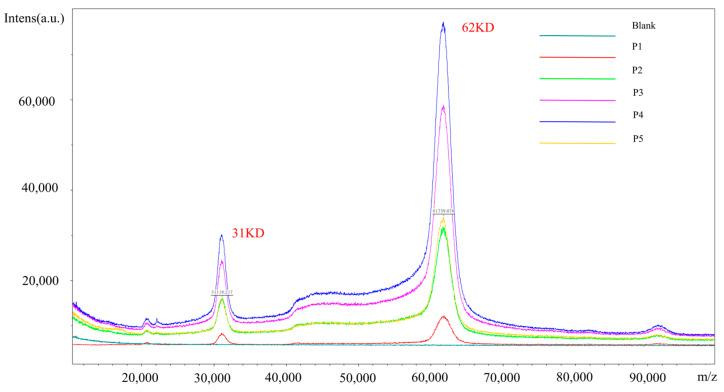
Molecular weight determination of collected isomers.

As shown in Figure 7, the measured molecular weights of the collected P1~P5 isomers were approximately 62 kDa and consistent with each other. The 62 kDa peak corresponds to the singly charged PEG-rhGH, while the 31 kDa peak represents the doubly charged species. These results confirm that the measured molecular weights are consistent with PEG-rhGH, ensuring the structural integrity of the isomers.

### 2.7. Potency Determination of PEG-rhGH Isomers

**Figure 8:**
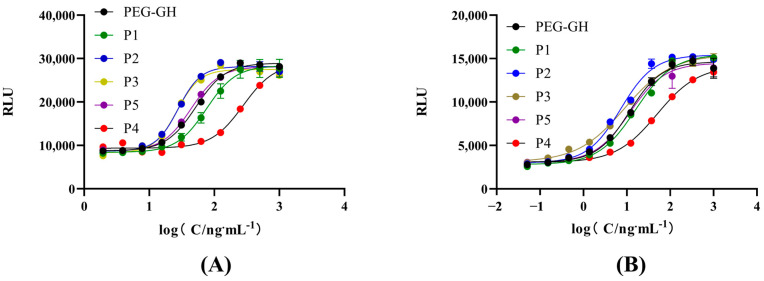
Dose–response curves of PEG-rhGH isomers and reference: (**A**) dose–response curves of PEG-rhGH isomers and reference sample using RGA; (**B**) dose–response curves of PEG-rhGH isomers and reference sample using NB2-11 cell proliferation assay.

As shown in Figure 8A, the bioactivity of P1 was slightly lower than the reference, reaching 71.13%. P2 and P3 exhibited significantly higher bioactivity levels, at 162.72% and 142.89%, respectively. In contrast, P4 showed markedly reduced bioactivity at 29%, while P5 displayed activity close to the reference, at 107.10%.

To further validate these findings, the Nb2-11 cell proliferation assay was conducted for each isomer. As shown in Figure 8B, the results aligned with those of the RGA. P1’s bioactivity was slightly lower than the reference at 75.12%, P5 was comparable to the reference at 97.40%, and P2 and P3 demonstrated significantly higher bioactivity levels at 168.06% and 144.40%, respectively. Conversely, P4 exhibited markedly reduced bioactivity at 26.35%.

The consistent results from both assays indicate that P2 and P3 have enhanced bioactivity, while P4 exhibits substantially reduced activity. These findings highlight that the biological activity of PEG-rhGH isomers depends on the modification site. Quality control of PEG-rhGH products should emphasize monitoring the proportion of these isomers. The reporter gene assay is a sensitive and reliable method for distinguishing isomer activity, making it an essential tool for PEG-rhGH quality control.

### 2.8. Structure–Activity Relationship Analysis of PEG-rhGH Isomers

According to previous studies, the major modification sites of PEG-rhGH have been identified as the N-terminus, K38, K140, K145, and K158. In this study, the isomer separation mechanism was shown to be consistent with the IEC method that this work used, and the modification sites of the different isomers were determined through trypsin digestion [14]. Therefore, based on the peak area percentages and peak shapes, the modification sites of the IEC-separated P1~P5 isomers were inferred to be the N-terminus, K140, K145, K38, and K158, respectively.

**Figure 9:**
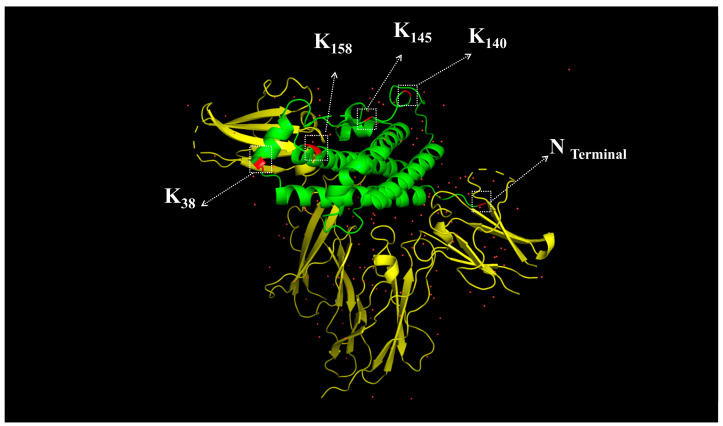
Structural alignment of the growth hormone receptor and rhGH.

After determining the modification sites of the different isomers, their structure–activity relationships were analyzed. In Figure 9, the yellow region represents rhGH, and the green region represents the growth hormone receptor. Our analysis revealed that the K38 amino acid on rhGH is very close to the GHR binding interface. Therefore, when K38 on the P4 isomer is PEGylated, the steric hindrance that is introduced by the large PEG molecule interferes with the binding of PEG-rhGH to GHR, affecting the downstream signal transduction, which likely explains the reduced bioactivity of this isomer compared to the reference. In contrast, K140 and K145 are positioned far from the GHR binding region, meaning that PEG modification at these sites does not significantly affect PEG-rhGH binding to GHR. Therefore, the P2 isomer (modified at K140) and P3 isomer (modified at K145) exhibit higher bioactivity levels than the reference. The modification sites at the N-terminus and K158 are farther from the receptor than K38 but closer than K140 and K145. Thus, the P1 isomer (modified at the N-terminus) and the P5 isomer (modified at K158) show higher bioactivity levels than P4 but lower than P2 and P3. This demonstrates that the bioactivity of the isomers is strongly correlated with their modification sites.

## 3. Discussion

This study developed an RGA based on the HepG2/IGF-1 cell line. The assay measures the amount of luciferase that is produced in live cells through luciferase reactions, indirectly reflecting the effect of PEG-rhGH on the HepG2/IGF-1 cell line and thereby assessing the biological activity of PEG-rhGH. The method offers high sensitivity, ease of operation, low variability, and good specificity, achieving results that are comparable to those obtained using the NB2-11 cell proliferation assay and in vivo animal testing.

In this study, ion exchange chromatography (IEX) was used to separate the PEG-rhGH isomers, followed by quantification of the isomers. After quantification, the activity of each isomer was assessed using both RGA and the NB2-11 assay. Previous studies have indicated that there is minimal activity variation among isomers when measured by animal assays [14]. Our results show that there are significant differences in the biological activity of the isomers, and their activity is closely related to the spatial structure of the isomers.

## 4. Materials and Methods

### 4.1. Cell Lines and Reagents

The EMEM culture medium was obtained from ATCC (Gaithersburg, MD, USA). The HepG2/IGF-1 cell was cultured in the EMEM medium, supplemented with 20% fetal bovine serum (FBS), 200 µg/mL hygromycin B, 2 µg/mL puromycin, and 5 µg/mL blasticidin S. All three antibiotics, lipofectamine 3000, recombinant human Interleukin-2 (IL-2, PHC0027), IL-4 (200-04), EGF (PHG0311L), and IGF-1 (RP-10931) were purchased from Thermo Fisher Scientific (Waltham, MA, USA). The Nb2-11 cell line was purchased from ECACC (UK). The Opti-MEM culture medium, FBS, and horse serum (HS) were purchased from Gibco (Grand Island, NY, USA). The TSKG3000 chromatographic columns used in this study were purchased from Tosoh, Japan, and the MALDI-TOF-MS instrument was acquired from Bruker, Bremen, Germany. The Steady-Glo^®^ Luciferase Assay System (E2520) and Cell Titer-Glo cell viability assay kit (G7571) were acquired from Promega, Madison, WI, USA, PEG-rhGH products and reference standard were supplied by GeneScience Pharmaceuticals Co. Ltd., Changchun, China and rhGH reference standards were acquired from the National Institutes for Food and Drug Control, Beijing, China.

### 4.2. Reporter Gene Assay Procedure

The cellular methodology was primarily established based on the method that is described in [28], with modifications that were tailored to the specific requirements of this study. The HepG2/IGF-1 cells were digested with 0.25% trypsin and rinsed once with PBS, followed by seeding into a 96-well white plate at a density of 8 × 10^5^ cells per well in 50 µL of the assay medium. The cells were then incubated at 37 °C with 5% CO_2_ overnight (16 to 24 h). The next day, the PEG-rhGH standard was serially diluted 2-fold in an assay medium at a starting concentration of 2 μg/mL. Then, 50 µL of diluted PEG-rhGH was added into each well. Notably, the final working concentration of PEG-rhGH was 1 μg/mL, and the final concentration ranged from 1000 ng/mL to 1.953 ng/mL. After 4 h of incubation, 100 µL of Steady-glo^®^ luciferase assay reagent was added into each well and mixed thoroughly under subdued light conditions for 15 min. The relative luminometer unit (RLU) signal was finally determined using a SpectraMax multimode plate reader after standing for another 15 min.

### 4.3. Optimization of Experimental Conditions for Reporter Gene Assay

The optimization of the RGA followed the same procedure as described in Section 4.2, with the only differences being the initial concentrations of the PEG-rhGH standard and the drug incubation time. In the optimization experiments, three different initial concentrations of PEG-rhGH were used: 1.0 μg/mL, 2.0 μg/mL, and 4.0 μg/mL. Additionally, the effects of different drug stimulation times were evaluated, including 2.5 h, 3 h, 3.5 h, 4 h, 5 h, and 6 h.

### 4.4. Methodological Validation of Reporter Gene Assay

#### 4.4.1. Accuracy, Precision, and Linearity of RGA

PEG-rhGH reference standards, used as the comparator, were prepared at initial concentrations of 1.0 μg/mL, 1.42 μg/mL, 2.0 μg/mL, 2.82 μg/mL, and 4.0 μg/mL and further diluted in two-fold increments to obtain a total of 10 concentration points. Each potency level was tested by two analysts, with each analyst performing four independent measurements. The biological activity determination followed the same procedure described in Section 4.1.

#### 4.4.2. Stability of RGA

We performed tests on both the 28th and 63rd generations of the HepG2/IGF-1 cells. The biological activity determination followed the same procedure that is described in Section 4.1.

#### 4.4.3. Stability and Potency Assessment of PEG-rhGH

PEG-rhGH samples were incubated at −20 °C for 0, 5, 10, 24, 36, and 72 h. Subsequently, the contents of aggregates and PEG-rhGH were analyzed using HPLC. According to the HPLC analysis method in the *Chinese Pharmacopoeia*, a size exclusion chromatography (SEC) column (300 × 7.8 mm) with a silica gel matrix was used as the stationary phase, with a flow rate of 0.6 mL/min and detection at a wavelength of 214 nm. The mobile phase consisted of 0.063 mol/L phosphate buffer and 3% isopropanol. Untreated and heat-treated PEG-rhGH samples (20 μL each) were injected into the column, and the relative content of each component was determined using the area normalization method.

The biological activity of the samples that were incubated at −20 °C was determined using the method that was described in Section 4.1, with the PEG-rhGH reference standard being used as the control.

### 4.5. Activity Measurement of Multiple Batches of PEG-rhGH and Consistency Verification with In Vivo Experiments

The in vitro activity of multiple batches of PEG-rhGH were determined using the method that was described in Section 4.2 and Section 4.6, with the PEG-rhGH reference standard being used as the control.

The in vivo bioassay of PEG-rhGH was performed in strict compliance with USP 40 General Chapter 126 for Multi-Batch Activity Determination.

For in vivo potency determination, male Sprague Dawley rats were hypophysectomized at 25–30 days of age. The rats were weighed 2 to 3 weeks after hypophysectomization, and only those that were in good health and had not gained or lost >10% of their preoperative body weight were used. Both the rhGH standard and the PEG-rhGH product samples were diluted with 0.1% bovine serum albumin to 0.2 IU/mL and 0.05 IU/mL before injection. The rats were randomly divided into 4 groups, each containing at least 8 rats. The rats were then injected subcutaneously with 0.5 mL of the standard solutions or sample solutions for 6 consecutive days. The body weight of each animal was recorded 24 h after the sixth injection. The change in body weight of each rat during the 6-day period was determined, and the strength of the sample solution relative to that of the standard solution was computed using parallel-line statistical analysis with Biostat software.Bland-Altman analysis was conducted to investigate the consistency of the two groups of data.

Bland-Altman analysis was conducted to investigate the consistency of RGA with in vivo and NB2-11 Cell Proliferation Assay.

### 4.6. In Vitro Nb2-11 Cell Proliferation Assay for PEG-rhGH Potency Measurement

Nb2-11 cells were cultured in Fischer’s medium supplemented with 10% heat-inactivated fetal bovine serum (FBS), 10% heat-inactivated horse serum (HS), 0.075% sodium bicarbonate, and 0.05 mM 2-mercaptoethanol under conditions of 37 °C and 5% CO₂. For the rhGH bioactivity measurement, Nb2-11 cells were suspended in Fischer’s assay medium (containing 1% heat-inactivated horse serum, 0.075% sodium bicarbonate, and 0.05 mM 2-mercaptoethanol) and seeded at 50,000 cells per well in 96-well plates, with a volume of 50 µL per well. Subsequently, multiple batches of PEG-rhGH reference standard samples were diluted in three-fold gradients, starting from an initial concentration of 1000 ng/mL, and 50 µL of each of the 10 different concentrations was added to each well. After 30 h of drug incubation, 100 μL of the Cell Titer-Glo Luminescent Kit was added to each well, followed by light-protected shaking for 30 min to allow for complete cell lysis. The relative luminescence units (RLUs) were then measured using a microplate reader. 

### 4.7. Separation, Sample Enrichment, and Peak Identification of PEG-rhGH Isomers

A BioPro IEX SF column was used to separate PEG-rhGH samples with different modification sites via their gradient elution. The flow rate was set to 0.4 mL/min, and detection was performed at a wavelength of 214 nm. Mobile phase A consisted of 0.01 mol/L acetate–acetate buffer (pH 4.90), while mobile phase B was composed of 0.01 mol/L acetate–acetate buffer with 0.3 mol/L sodium chloride (pH 4.90). The total run time was 80 min, with the proportion of mobile phase B starting at 2%, increasing to 10% at 60 min, and then returning to 2% at 61 min. The collected isomers were concentrated using a 3 kDa ultrafiltration centrifuge tube.

The collected isomer samples were immediately subjected to Peak Identification using the same chromatographic method as employed during the separation.

### 4.8. Quantification of Collected Isomers

The collected samples were subjected to isocratic elution using a TSKgel G3000SWxL (from Tosoh, Tokyo, Japan) column to determine their content. The flow rate was set to 0.6 mL/min, with detection at a wavelength of 214 nm. The mobile phase consisted of 0.5% triethylamine in 0.05 mol/L phosphate buffer (pH 7.0). The sample temperature was controlled at 6 °C, with an injection volume of 20 μL, and the elution time was set to 30 min. The components and contents of the samples were analyzed using a UV detector for subsequent bioactivity assays using the following formula: Isomer Concentratiosomer mian Peak Area/PEG-rhGH reference standard mian Peak Area*reference standard concentration.

### 4.9. Molecular Weight Determination

The collected isomers were diluted to 1 mg/mL and mixed with SA (sinapinic acid) at a 1:1 ratio. The mixed samples were then spotted onto a MALDI target plate for analysis. Using MALDI-TOF-MS, we determined the molecular weights of the isomers.

### 4.10. Potency Determination of PEG-rhGH Isomers

To investigate differences in the biological activity levels of the PEG-rhGH isomers, the isomers and the PEG-rhGH reference standard were diluted to 2 μg/mL, and their bioactivity was measured using the RGA, following the same procedure as described in Section 4.2.

## 5. Conclusions

This study successfully established an in vitro bioactivity assay for PEG-rhGH using the HepG2/IGF-1 reporter gene system. The assay demonstrated high sensitivity, simplicity, low variability, and good reproducibility. Key experimental parameters were optimized, including an initial PEG-rhGH concentration of 2 μg/mL with 1:1 serial dilutions, seeding at 8 × 10^5^ cells per well, and a 4 h stimulation time. The validation confirmed good accuracy and precision across a potency range of 1 μg/mL (50%) to 4 μg/mL (200%).

Compared to the Nb2-11 assay, the Reporter Gene Assay (RGA) not only reduced the detection time but also directly assessed downstream signaling pathway activation following PEG-rhGH binding to GHR, making it more mechanistically relevant. When applied to analyze PEG-rhGH isomers with different modification sites, the RGA revealed significant bioactivity differences, which was consistent with results from the Nb2-11 cell proliferation assay.

This method provides a reliable tool for quality control of PEG-rhGH, enabling the evaluation of bioactivity differences among modification sites and ensuring product quality and safety.

## Figures and Tables

**Table 1:** Results of the validation of accuracy and precision.

Sample Concentration (μg/mL)	Mean of Measured Potency %	Recovery %	Intermediate Precision (GCV%)	Relative Bias %
1	49.82	99.64	2.1	−0.4
1.42	68.52	96.50	5.0	−3.6
2	93.57	93.57	7.4	−6.6
2.82	138.77	98.42	3.9	−1.6%
4	196.58	98.29	4.2	−1.8%

**Table 2:** Potency measurement of 9 batches of PEG-rhGH.

Sample Batch	Potency Analyis by NB2-11 (IU/mg)	Potency Analyis by RGA (IU/mg)	Potency Analyis by In Vivo Assay (IU/mg)
2022050219	7.25	7.83	6.59
2022050226	7.12	7.81	6.47
2022060249	7.00	7.68	6.62
2022050219	7.10	7.64	6.69
2023050327	6.62	7.60	5.36
2023050310	7.31	8.30	6.34
2022050196	7.55	7.56	6.32
2023040188	7.29	6.71	5.51
2022120717	7.07	6.88	5.41

**Table 3:** Quantitative determination of P1~P5 isomers.

Sample	Main Peak Area (*n* = 4)	Concentration (mg/mL)
PEG-rhGH	16,957,970	0.985
P1	16,497,058	0.958
P2	14,868,422	0.864
P3	16,659,860	0.967
P4	16,119,476	0.936
P5	16,191,786	0.940

## Data Availability

Data are contained within the article.
